# Serum CGRP Changes following Ultrasound-Guided Bilateral Greater-Occipital-Nerve Block

**DOI:** 10.3390/neurolint14010016

**Published:** 2022-02-07

**Authors:** Abdelrahman Abbas, Ramez Moustafa, Ali Shalash, Mahmoud Haroun, Randa Amin, Sherien Borham, Ahmed Elsadek, Shahinaz Helmy

**Affiliations:** Department of Neurology, Faculty of Medicine, Ain Shams University, 12th Abbassiya Street, Cairo 08080, Egypt

**Keywords:** CGRP, GONB, chronic migraine

## Abstract

Background: Calcitonin-gene-related peptide (CGRP) and CGRP receptors are expressed in trigeminal nerve cells, and treatments targeting CGRP are effective in migraines. For headaches that do not respond to pharmacological treatment, minimally invasive techniques such as greater-occipital-nerve block (GONB) can help relieve the pain and reduce the frequency of headaches. Our study assessed the efficacy of ultrasound-guided greater-occipital-nerve block (USgGONB) in chronic migraines (CM) and its relationship to serum CGRP levels. Methods: Forty chronic migraineurs who underwent bilateral USgGONB using 40 mg triamcinolone and 1 mL lidocaine were recruited and interictal serum CGRP samples were collected immediately before and one month after GONB. The clinical response was evaluated using headache diaries before and one month after USgGONB. The patient response was determined after USgGONB according to the reduction in headache days as a good responder (>50% reduction), poor responder (<50%) or non-responder. Results: Monthly headache days after GONB showed a significant reduction (median, 10 days; range, 8–14.7) compared to before the block (median, 18 days; range, 17–22; *p* < 0.001). Across all patients, interictal serum CGRP levels after USgGONB were significantly lower than before the block (median, 40 pg/mL (range, 25–60) vs. 145 pg/mL (range, 60–380) (*p* = 0.001). The pre-treatment interictal CGRP levels showed a significant difference (*p* = 0.003), as their levels in non-responders (median, 310 pg/mL; interquartile range, 262–350) were significantly higher than those seen in responders, whether poor responders (median, 135 pg/mL; interquartile range, 100–200 pg/mL) or good responders (median, 140 pg/mL; interquartile range, 80–150 pg/mL). Conclusion: the study showed the beneficial effect of USgGONB in chronic migraines that was associated with lowering interictal CGRP levels, implying a potential role for CGRP in the mechanism of action of GONB in CM, and the interictal CGRP level may be used as a predictor for the response to GONB.

## 1. Introduction

Migraine is a prevalent, multifactorial neurovascular disorder that has significant personal and societal consequences [1]. Migraines impact approximately 15% of the population for up to three days of debilitating symptoms such as severe headaches and vomiting as well as an extreme sensitivity to light, sound, and smell [2].

In chronic migraines there is a state of imbalance between cortical hyper-excitability and normal habituation, and an increased preictal facilitation of the control of inhibitory mechanisms [3]. Moreover, neurophysiological studies on animal models of familial hemiplegic migraines revealed the upregulation of cortical glutamatergic circuits, which is considered a critical pathophysiological mechanistic basis underlying the migraine [4]. These findings were supported by neurophysiological studies and were described as thalamocortical dysrhythmia [5].

This imbalance between cortical responsivity and sensory processing coupled with a mismatch between the brain’s energy reserve and workload may stimulate the trigeminovascular system, which surrounds the cranial blood vessels with nociceptive trigeminal sensory afferents [6]. Neurons in the trigeminocervical complex receive signals from these perivascular trigeminal afferents via the neurotransmitter Calcitonin-gene-related peptide (CGRP) [7]. The trigeminal nucleus caudalis (TNC) and the C1–2 level of the spinal cord are thought to be affected by CGRP, which transmits pain signals to the thalamus and higher cortical pain regions [8].

In migraine sufferers, the systemic administration of CGRP induces a migraine-like attack that is phenotypically equivalent to the subject’s spontaneous attack [9]. Additionally, CGRP levels are increased throughout the migraine attacks and gradually increase in chronic-migraine patients on a continuous basis. The new anti-CGRP medications have shown great efficacy in the treatment of migraines of all subtypes, increasing the evidence that CGRP plays an important role in migraine pathophysiology [10].

Numerous studies have established the safety and effectiveness of peripheral-nerve blocks in the treatment of a wide range of headache disorders. Migraines may become resistant to conventional pharmacologic treatment, and in these patients, minimally invasive methods including peripheral-nerve blocks may be employed to relieve pain and reduce attack incidence. These techniques also help to decrease the need, and hence the systemic side effects of pharmacological therapy [11].

The onabotulinum toxin is a locally injected medication using a minimally invasive technique that has shown significant effectiveness in chronic migraines for more than a decade since its approval for treatment of this condition. Interestingly, it has been shown that the response to onabotulinum toxin is associated with a reduction in serum CGRP in chronic-migraine patients [12]. Similarly, greater-occipital-nerve block (GONB) was demonstrated to decrease pain intensity and analgesic-therapy intake, as well as the total number of headaches in migraine patients within one week of intervention, with no boost in adverse outcomes [13]. Ultrasound guidance increases the performance of GONB and may enable a more accurate nerve block [14]. However, it is unknown if the benefit of GONB is also associated with the modulation of CGRP.

The current study assessed the efficacy of ultrasound-guided greater-occipital-nerve block (USgGONB) in chronic migraine (CM) and its relationship to interictal serum CGRP levels.

## 2. Materials and Methods

The study sample was based on the study carried out by Cernuda-Morollón et al. [8]. We used Epi Info STATCALC (Atlanta, GA, USA, 2018) to calculate the sample size of 40 patients then we consecutively recruited 40 participants that had been diagnosed with CM from the headache outpatient clinics at the Department of Neurology, Ain Shams University Hospitals (Cairo, Egypt) between August 2018 and March 2021. This was an observational study with a convenience sample.

The inclusion criteria were patients with chronic migraines above 18 years of age. Written informed consent was obtained from all participants and the study was approved by Ain Shams University Ethics Committee (approval number FWA 000017585). Exclusion criteria were patients with severe systemic diseases, infection or injury to the occiput and patients with allergy to any of the substances used in the injection. All patients were clinically evaluated (detailed history including personal data, medical history, and migraine treatments used). All patients underwent ultrasound-guided bilateral GONB with 40 mg triamcinolone and 1 mL 2% lidocaine using a portable ultrasound system with a 7–13 MHz multifrequency transducer (ACUSON Juniper Ultrasound System, Siemens, Germany).

Blood samples were collected after an overnight fast before the procedure and after one month. Samples were collected between 9 and 11 am to avoid the effect of circadian rhythms on CGRP level. Subjects were to stop using any anti-inflammatory or analgesic medication in the previous 48 h. The blood sample was taken from the non-dominant forearm to measure interictal serum CGRP levels using commercial ELISA kits (Novus Biologicals Inc., USA) following the manufacturer’s instructions. Absorption levels were measured with a spectrophotometer at a wavelength of 450 nm ± 2 nm. The detection limit of the assay was 9.3 pg/mL for CGRP.

Clinical efficacy was assessed using migraine diaries completed by patients over one month following the blockade. The patient response was determined according to the reduction in headache days after GONB as follows: good responder > 50% reduction, poor responder < 50% reduction or non-responder if there was no change at all.

Statistical analysis: The collected data were revised, coded and entered onto a personal computer utilizing the Statistical Package for Social Science (SPSS version 25, IBM: Armonk, NY, USA). Analysis was performed according to the type of data obtained for each parameter; mean and standard deviation (±SD) for parametric numerical data, while median and interquartile range (IQR) were used for non-parametric numerical data. Frequency and percentage were used for non-numerical data. The Chi-Square test was used to determine the relationship between two qualitative variables. The Wilcoxon Signed Ranks Test was used to investigate the statistical significance of the difference between the two study-group medians. The Kruskal–Wallis Test was used to determine the statistical significance of the difference between more than two study-group medians. A receiver-operator-characteristics analysis was performed to identify the cut-off value of the CGRP predicting response to GONB. *p* values greater than 0.05 were regarded as non-significant, those less than 0.05 as significant, and those less than 0.01 as highly significant.

## 3. Results

### 3.1. Clinical Data

Forty patients fulfilling the criteria for chronic migraine (CM) were enrolled in this study. The mean age of patients was 31.1 ± 7.3. Only nine (22.5%) were males while the remainder (*n* = 31; 77.5 percent) were females. The average duration that patients had CM was 8 ± 2.8 years. Table 1 summarizes the major phenotypes and associated symptoms of the patients recruited in this study.

### 3.2. Clinical Response after GONB

**Figure 1:**
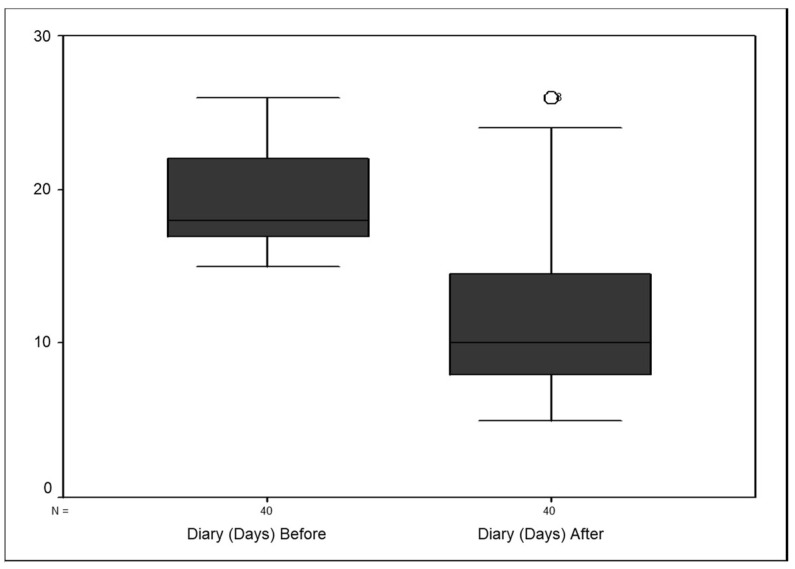
Box plot showing reduction in headache days after GONB (*n* = 40).

Monthly headache days after GONB showed a significant reduction (median, 10 days; range, 8–14.7) compared to before the block (median, 18 days; range, 17–22; *p* < 0.001) as shown in Figure 1.

Thirty-four patients (85%) responded to GONB and the remaining six patients (15%) did not notice any response. Sixteen patients (40%) showed a good response while eighteen patients (45%) showed poor response, and there were no side effects reported from the procedure apart from mild injection-site pain in about twenty-four patients.

### 3.3. CGRP Determinations and Difference with Clinical Response

**Figure 2:**
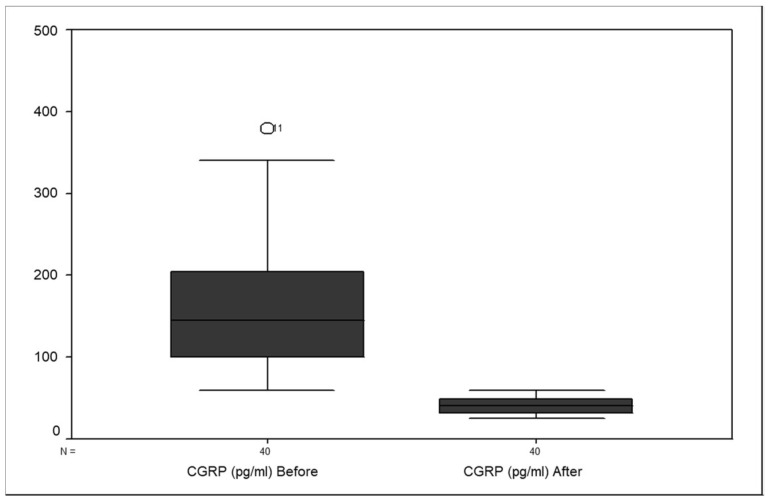
Box plot for interictal CGRP levels in chronic-migraine patients before and after GONB (*n* = 40).

- The interictal CGRP levels were significantly lower in all patients following GONB (median, 40 pg/mL; range, 25–60) than they were prior to GONB (median, 145 pg/mL; range, 60–380; *p*-value = 0.001) as shown in Figure 2.
- The interictal CGRP levels before the injection response showed a significant difference (*p* = 0.003), as the pre-treatment CGRP levels in non-responders (median, 310 pg/mL; range, 262–350 pg/mL) were significantly higher than those seen in the responders, whether poor responders (median, 135 pg/mL; range, 100–200 pg/mL) or good responders (median, 140 pg/mL; range, 80–150 pg/mL).
- The post-treatment interictal CGRP levels in both non-responders (median, 44 pg/mL; range, 38.75–50 pg/mL) and responders, whether poor responders (median, 41 pg/mL; range, 31–45.75 pg/mL) or good responders (median, 36 pg/mL; range, 32.25–50) one month after treatment showed no significant difference (*p* = 0.577) as shown in Table 2.

### 3.4. ROC Curve Analysis to Detect the Response Prediction Accuracy of Pre-GONB Interictal CGRP Level in CM

**Figure 3:**
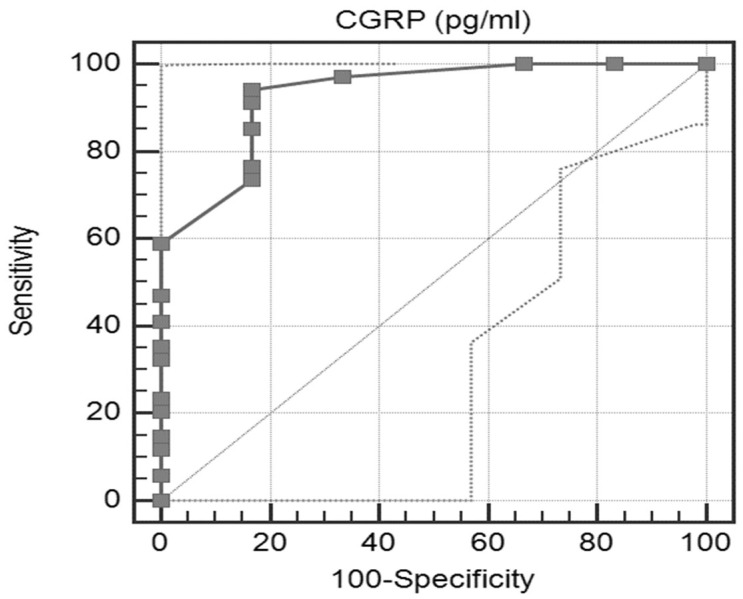
ROC curve of pre-GONB CGRP level.

By using the ROC-curve analysis, the CGRP level at a cutoff point of ≤250 pg/mL predicted patients that were responsive to GONB, with fair accuracy (93.1%), sensitivity (94.1%) and specificity (83.3%) as shown in Figure 3.

## 4. Discussion

Elevated serum CGRP has been suggested as a biomarker to facilitate a more objective diagnosis of CM in cases with frequent or almost daily headaches and a background of migraines [10]. The present study demonstrated that treatment with GONB significantly reduces serum interictal CGRP levels in patients with CM. These findings will lead to a better understanding of GONB’s mode of action in CM and the suggestion that interictal CGRP levels may aid in forecasting the response to GONB.

In the majority of patients, our study revealed a significant decrease in headache days before and after GONB. Similar findings were discovered in a study conducted by Ashkenazi et al. [15]. In contrast, an earlier study demonstrated that GON blockade was evaluated for its diagnostic value and effect and found that only 6% of the migraine group showed a good response, which may be attributed to the blockade type as they used 0.5 to 1.5 mL of 2% lidocaine with a 12.5 μg/mL adrenaline injection. Additionally, using the ultrasound guided technique in our present study probably added more accuracy and precise localization of the nerve, which improved the responses among our patients [16].

The current study demonstrated that the CGRP levels were comparable to other studies and relatively higher prior to GONB, which is similar to a study in which they assessed plasma specimens from 103 women with CM (all over the age of 17 years) and 31 matched healthy women with no historical record of headache, 43 paired women with episodic migraines (EM), and 14 matched cases with episodic cluster headaches. In comparison to healthy controls, the women with EM, the patients with episodic cluster headache, and the CM group had higher CGRP levels [9].

Importantly, the current study showed a significant reduction in CGRP levels after GONB. There are studies that measured CGRP-level changes after botulinum toxin injection such as that of Cernuda-Morollón et al. [12], who looked at whether treatment with onabotulinum toxin A (onabotA) was able to cause alterations in interictal plasma CGRP concentrations. They showed that serum CGRP levels were high in patients with CM at a baseline that significantly decreased following onabotA treatment [12]

In our study we found that 45% of the patients showed a good response, 40% showed a poor response and about 15% showed no response at all. We showed not only that CGRP levels are reduced after GONB, but also that the pre-treatment CGRP levels may predict the response to treatment. CGRP was high in responders but was markedly higher in non-responders (*p* = 0.003). In our assay, we found that CGRP levels before GONB may play a role as a potential predictor for the outcome of the GONB, as a level of CGRP exceeding 250 pg/mL showed no response while lower levels than 250 pg/mL showed response ranging from poor (<50%) to good response (>50%). Nonetheless, both responders and non-responders showed a significant reduction in CGRP levels, which may raise the importance of repeated GONB in non-responders as found by Inan et al. [17], who performed repeated GON blocks four times weekly and showed a considerable minimization of the number of headaches and visual-analogue-scale (VAS) scores.

In our study, we found that the post-treatment CGRP levels were not significant between groups (*p* = 0.57). This raises the possibility that clinical improvement may not be entirely CGRP-dependent. The findings in non-responders may also be explained by the study’s limitations, which include the absence of a control group, the fact that the majority of these patients remained on oral preventives, and the low number of non-responders. Moreover, because a migraine is intrinsically and subjectively variable, some patients with a “CM” phenotype may have other headaches (e.g., psychogenic, tension-type headache or other secondary headaches) to which CGRP does not seem to be related. This may be because pain may be secondary to a predominance of parasympathetic stimulation of the trigeminovascular system (TVS) in some migraine patients [18]. For example, the efficacy of pure CGRP antagonists dubbed “gepants” tends to be shorter than that of triptans, which are recognized to inhibit both CGRP and VIP secretion by the TVS [2].

Our study is limited by the small number of patients and the lack of a control group for comparison. Nonetheless, as a pilot study, it provides evidence of the role of CGRP in local intervention in this group of patients.

## 5. Conclusions

This is, to our knowledge, one of the first studies to assess the changes of CGRP levels with GONB, particularly using ultrasound guidance and detailed follow-up of outcome. Our results suggest a beneficial role of GONB in chronic migraines that is reflected in interictal serum CGRP levels. Furthermore, very high pre-treatment CGRP levels may predict an unfavorable response to GONB.

## Figures and Tables

**Table 1:** Summary of clinical characteristics of our patients with chronic migraines.

Phenotypes and Associated Symptoms		N	%
Laterality	Unilateral	18	45
	Bilateral	22	55
Area	Frontal	7	17.50
	Temporal	23	57.50
	Occipital	10	25.00
Photophobia	No	7	17.50
	Yes	33	82.50
Phonophobia	No	5	12.50
	Yes	35	87.50
Lacrimation	No	18	45
	Yes	22	55
Vertigo	No	17	42.50
	Yes	23	57.50

**Table 2:** Interictal CGRP levels before and after GONB and its difference among response groups.

		Median	IQR	Kruskal-Wallis Test	
				X^2^	*p*-Value
CGRP (pg/mL) Before	No Response	310	262.5–350	11.839	0.003 *
	Poor Response	135	100–200		
	Good Response	140	80–150		
CGRP (pg/mL) After	No Response	44	38.75–50	1.099	0.577
	Poor Response	41	31–45.75		
	Good Response	36	32.25–50		

* Means *p* value is significant.

## Data Availability

All the data that support the findings of this research are available from the corresponding author Ramez Moustafa upon reasonable request.
